# Risk factors, complications and survival after upper abdominal surgery: a prospective cohort study

**DOI:** 10.1186/s12893-015-0069-2

**Published:** 2015-07-07

**Authors:** E. K. Aahlin, G. Tranø, N. Johns, A. Horn, J. A. Søreide, K.C. Fearon, A. Revhaug, K. Lassen

**Affiliations:** Department of GI and HPB Surgery, University Hospital of Northern Norway, Tromsø, 9038 Breivika Norway; Institute of Clinical Medicine, University of Tromsø - The Arctic University of Norway, Tromsø, Norway; Department of Gastrointestinal Surgery, St. Olavs Hospital - Trondheim University Hospital, Trondheim, Norway; Clinical Surgery, University of Edinburgh, Royal Infirmary of Edinburgh, Edinburgh, UK; Department of Abdominal and Emergency Surgery, Haukeland University Hospital, Bergen, Norway; Department of Gastrointestinal Surgery, Stavanger University Hospital, Stavanger, Norway; Department of Clinical Medicine, University of Bergen, Bergen, Norway

**Keywords:** Sarcopenia, Cachexia, Cancer, Gastrointestinal, Hepatopancreatobiliary, Surgery

## Abstract

**Background:**

Preoperative weight loss and abnormal serum-albumin have traditionally been associated with reduced survival. More recently, a correlation between postoperative complications and reduced long-term survival has been reported and the significance of the relative proportion of skeletal muscle, visceral and subcutaneous adipose tissue has been examined with conflicting results. We investigated how preoperative body composition and major non-fatal complications related to overall survival and compared this to established predictors in a large cohort undergoing upper abdominal surgery.

**Methods:**

From 2001 to 2006, 447 patients were included in a Norwegian multicenter randomized controlled trial in major upper abdominal surgery. Patients were now, six years later, analyzed as a single prospective cohort and overall survival was retrieved from the National Population Registry. Body composition indices were calculated from CT images taken within three months preoperatively.

**Results:**

Preoperative serum-albumin <35 g/l (HR = 1.52, p = 0 .014) and weight loss >5 % (HR = 1.38, *p* = 0.023) were independently associated with reduced survival. There was no association between any of the preoperative body composition indices and reduced survival.

Major postoperative complications were independently associated with reduced survival but only as long as patients who died within 90 days were included in the analysis.

**Conclusions:**

Our study has confirmed the robust significance of the traditional indicators, preoperative serum-albumin and weight loss. The body composition indices did not prove beneficial as global indicators of poor prognosis in upper abdominal surgery. We found no association between non-fatal postoperative complications and long-term survival.

## Background

Preoperative weight loss and abnormal serum-albumin have been associated with unfavorable postoperative outcome for more than a decade [1–3]. An involuntary weight loss of five percent or more has been recognized to be associated with reduced survival for more than thirty years and it has been proposed as a diagnostic criterion for cancer cachexia [4–6].

Sarcopenia (i.e. loss of skeletal muscle) has been shown to be associated with reduced survival after cancer surgery [7–9]. Sarcopenia has also been associated with postoperative complications [10], increased chemotherapy toxicity and decreased time to tumor progression [11]. While visceral adiposity has been associated with diabetes, and diabetes again has been associated with unfavorable outcome after surgery; evaluations of a direct association between adipose tissue indices and unfavorable outcome have shown conflicting results [9, 12, 13].

An association between major postoperative complications and reduced long-term survival has been shown for a wide range of malignant and non-malignant diseases [14–19]. Khuri et al. reported such association even when those who die within 30 days after surgery were excluded from analysis and it has been suggested that complications could alter the immune system and make patients more susceptible to cancer recurrence [14, 15]. In light of improved, modern intensive care medicine we suspect that a 30 day cut-off might not correctly identify those who die as a direct result of their complication. It is not known whether there is an association between complications and long-term survival in major upper abdominal surgery when only those who survive more than 90 days after surgery are analyzed.

We aimed to investigate the association between preoperative body composition indices, weight loss and serum-albumin, postoperative complications and overall survival after major upper abdominal surgery.

## Methods

From 2001 to 2006, 447 patients were included in a Norwegian multicenter randomized controlled trial (RCT), which investigated normal food at will after major upper gastrointestinal (GI) and hepatopancreatobiliary (HPB) surgery [20]. Further analysis has not been performed on this dataset and it was treated here as a single, prospective cohort. We retrieved survival data from the Norwegian Population Registry during April 2012, six years after the last patient was included in the original trial.

In addition to demographics and general clinical characteristics, the prospective database included specifically: patient-reported preoperative weight loss, preoperative serum-albumin, type of surgery and major postoperative complications. Major complications, within eight weeks after surgery where defined a priori in the original trial [20] (Table 1). The operative procedures were listed in the original publication [20].

**Table 1:** Major postoperative complications: definition and criteria

Complication	Criteria
SIRS	Two or more of the following:
	Temperature >38 °C or <36 °C
	Heart rate >90 beats/min
	Respiratory rate >20/min or PaCO2 < 4,3kPa
	White blood cell count >12000 cell/ml or
	<4000 cells/ml or >10 % immature forms
Bacteraemia	At least one positive blood culture of pathogenic organisms
Sepsis	SIRS + bacteraemia
Pneumonia	X-ray confirmed and necessitating antibiotic treatment
Anastomotic leak	Necessitating re-operation or demonstrated upon autopsy
Bowel obstruction, necrosis or perforation	Necessitating re-operation or demonstrated upon autopsy
Intra abdominal abscess	Necessitating re-operation or percutaneous drainage or demonstrated upon autopsy
Intra abdominal haemorrhage	Necessitating re-operation, transfusion of six or more units of Packed Red Blood Cells within first 48 h postop. or demonstrated upon autopsy
Wound rupture	Necessitating re-suturing in general anesthesia or demonstrated upon autopsy
Pancreatitis	Serum enzyme level twice upper normal value, absence of known pre-existing pancreatitis, ERCP or mechanical trauma to pancreas during operation providing satisfactory explanation for elevated enzymes
Cholecystitis	Confirmed by histological examination of specimen or by cultured content from percutaneous drainage
Myocardial infarction	Diagnostic enzyme pattern and either typical pain or ECG changes, or demonstrated upon autopsy
Myocardial arrhythmia	ECG confirmed, with hypotension or symptomatic angina, necessitating stabilizing drugs or electro-conversion
Cardiac arrest	Confirmed by ECG or cardiac rhythm monitoring, and necessitating resuscitation
Acute Cardiac failure	Confirmed by by Echocardiosonography or necessitating pressure agents
Cerebrovascular infarction or cerebrovascular haemorrhage	New, and persistent (>48 h) central neurological deficit, and confirmed by CT scan or demonstrated upon autopsy
Pulmonary embolism	Confirmed by unequivocal nuclear isotope scan, ECG, pulmonary angiography, spiral technique CT scan or demonstrated upon autopsy
Pulmonary insufficiency	Necessitating postoperative ventilation support more than 24 h

Major postoperative complications as defined in the original randomized trial [20]

Preoperative weight loss was calculated from patients’ usual pre-morbid weight as they reported it - with no time limit, and current weight as scaled preoperatively at trial enrolment. We used well-established cut-offs for preoperative weight loss and serum-albumin [6, 21]. Preoperative weight loss was dichotomized into >5 % or ≤5 %. Preoperative serum-albumin was dichotomized into <35.0 g/l or ≥35.0 g/l. Body Mass Index, BMI was calculated with the following formula [22]: BMI = weight (kg)/height^2^ (m).

Digitally stored computer tomography (CT) images for initial routine diagnostics and staging were analyzed using Slice-O-Matic software V4.2 (Tomovision, Montreal-Canada) which permitted specific tissue demarcation using Houndsfield unit threshold of −29 to +150 for skeletal muscles [23], −150 to −50 for visceral adipose tissue [24], and −190 to −30 for subcutaneous adipose tissue [23]. Cross-sectional areas (cm^2^) were calculated for each tissue by summing tissue pixels and multiplying by the pixel surface area. A transverse CT image from the third lumbar vertebrae (L3) was assessed for each scan and tissue areas estimated [25]. All CT images were analyzed by one single trained observer who was blinded to all clinical data. Cross-sectional area was normalized for stature (cm^2^/m^2^) and the following indices were calculated: L3 Skeletal muscle index (L3 SMI), L3 Visceral adipose tissue index (L3 VAT) and L3 Subcutaneous adipose tissue index (L3 SAT). CT images used for analysis were retrieved from routine images done within three months preoperatively.

We used the cut-off for L3 SMI suggested by Martin et al. [26]: L3 SMI <41 cm^2^/m^2^ for women, L3 SMI <43 cm^2^/m^2^ for men with BMI <25 kg/m^2^ and L3 SMI <53 cm^2^/m^2^ for men with BMI ≥25 kg/m^2^. We also calculated the percentage of women and men with L3 SMI lower than the cut-offs suggested by Mourtzakis et al. and later used in a consensus article on cachexia and sarcopenia [6, 27], which corresponds to skeletal muscle mass two standard deviations from that of healthy young adults (39 cm^2^/m^2^ for women and 55 cm^2^/m^2^ for men). Preoperative weight loss, serum-albumin and L3 SMI were analyzed both as dichotomous and continuous variables. L3 VAT and L3 SAT were analyzed only as continuous variables.

All patients with complete data sets for preoperative serum-albumin and weight loss were included in analysis. A subgroup analysis was performed on patients with available preoperative CT images. Patients were divided into the following disease-categories: Gastroesophageal cancer, pancreatic cancer, other cancer (mainly malignant liver tumors and colorectal liver metastasis) and non-cancer.

### Statistics

Statistical analysis was performed with SPSS statistics software, version 22 (IBM, New York -USA). A Cox proportional hazard regression analysis, stratified for disease-categories, was used for analysis of overall survival; the assumption of proportional hazards was visually inspected by log-log survival curves. For comparison of characteristics between different groups and categories, analysis of variance (ANOVA) for continuous data and Pearson chi-square test for categorical data were used. Kruskal-Wallis test and Mann–Whitney *U* test were used for non-normally distributed data. P-values <0.05 were considered statistically significant.

## Results

### Selection and characteristics

There were 447 patients included in the original trial. Survival data were obtained for 438 patients (98.0 %). Complete information on preoperative weight loss and serum-albumin was available for 369 patients (82.5 %). Preoperative CT images of sufficient quality were available in 157 of these patients (157/369 = 42.5 %). There were no significant differences in complication rates or overall survival between patients included (*n* = 369) and those excluded due to missing nutritional data (*n* = 69). There were no significant differences in complication rates or overall survival between patients with available CT images (*n* = 157) and patients without available CT images (*n* = 212). Of the 369 patients available for analysis, 26 patients (7.0 %) had died within 90 days. The five year mortality rates varied between 20.4 % in patients without cancer and 77.6 % in patients with pancreatic cancer (Table 2).

**Table 2:** Disease categories: characteristics, postoperative complications and 5-year mortality

	Gastroesophageal cancer	Pancreatic cancer	0ther cancer	Non cancer	*P*-value
	(*n* = 122)	(*n* = 85)	(*n* = 64)	(*n* = 98)	
Age, mean (SD)	68 (13)	64 (10)	63 (13)	60 (15)	<0.001
Male gender, n (%)	75 (61.4 %)	58 (68.2 %)	36 (56.3 %)	50 (51.0 %)	0.108
Complications^a^, n (%)	45 (36.9 %)	27 (31.8 %)	21 (32.8 %)	22 (22.4 %)	0.143
5-year mortality, n (%)	79 (64.8 %)	66 (77.6 %)	35 (54.7 %)	20 (20.4 %)	<0.001

^a^) Major postoperative complications within eight weeks after surgery

### Preoperative weight loss, serum-albumin and body composition

Median preoperative weight loss was 1.8 % (not normally distributed). There were 131 patients with preoperative weight loss >5 % (35.6 %) while 175 patients had no preoperative weight loss (47.4 %). Mean serum-albumin concentration was 39.0 g/l. The number of patients with serum-albumin <35 g/l was 62 (16.8 %).

Five-year mortality spanned from 44.6 % in patients with no preoperative weight loss to 67.8 % in patients with >10 % preoperative weight loss (Table 3). There were no statistically significant differences in L3 SMI between different preoperative weight loss categories (Table 3).

**Table 3:** Categories of preoperative weight loss: characteristics, complications and 5-year mortality

	No weight loss	1-5 % weight loss	6-10 % weight loss	>10 % weight loss	*P*-value
	(*n* = 175)	(*n* = 63)	(*n* = 72)	(*n* = 59)	
Serum-albumin (g/l), mean (SD)	40.3 (5.6)	38.4 (5.6)	38.1 (4.4)	36.9 (5.4)	<0.001
L3 SMI, mean (SD)^a^	45.6 (7.3)	43.1 (5.5)	46.6 (5.6)	43.0 (6.1)	0.071
Complications^b^	51 (29.1 %)	21 (33.3 %)	25 (34.7 %)	18 (30.5 %)	0.823
5-year mortality, n (%)	78 (44.6 %)	37 (58.7 %)	45 (62.5 %)	40 (67.8 %)	0.004

^a)^ Calculated for patients with available preoperative CT images only (*n* = 157)

^b)^ Major postoperative complications within eight weeks after surgery

Mean L3 SMI was 41.8 cm^2^/m^2^ in women and 47.0 cm^2^/m^2^ in men. Of the 157 patients with L3 SMI analyzed, 72 (45.9 %) had L3 SMI below Martin's cut-off [26]. Only 7 (9.7 %) of these patients were obese. The proportion of women and men with L3 SMI below Martin’s cut-off were 47.5 % and 44.8 % respectively.

**Fig. 1:**
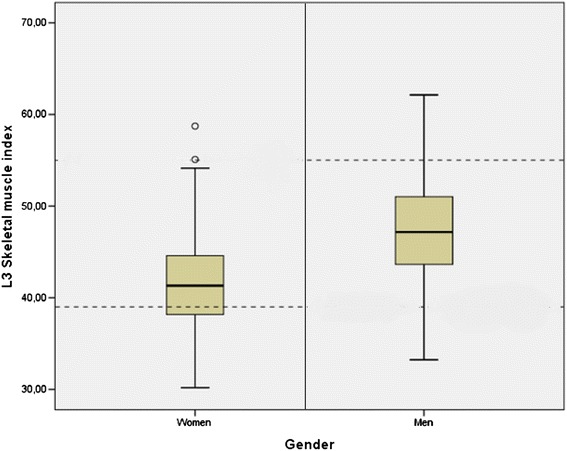
Distribution of L3 skeletal muscle index

The number of patients with L3 SMI below Mourtzakis’s cut-off [27] was 110 (70.1 %). Only 6 (5.5 %) of these patients were obese. The proportion of women and men with L3 SMI below Mourtzakis’s cut-off were 37.7 % and 90.6 % respectively (Fig. 1).

A significant difference in BMI and age by quartile categories of L3 SMI was observed (Table 4). However, no significant differences in complication rates or five-year mortality between the different quartile categories of L3 SMI were found, nor did we demonstrate any differences in mean preoperative weight loss or serum-albumin (Table 4).

**Table 4:** Quartile categories of L3 Skeletal muscle index: characteristics, complications and 5-year mortality

	1. quartile (*n* = 39)	2. quartile (*n* = 39)	3. quartile (*n* = 36)	4. quartile (*n* = 43)	*P*-value
	Range L3 SMI (cm^2^/m^2^) = W: 30.2-38.1	Range L3 SMI (cm^2^/m^2^) = W: 38.2-41.1	Range L3 SMI (cm^2^/m^2^) = W: 41.3-44.5	Range L3 SMI (cm^2^/m^2^) = W: 45.6-58.7	
	M: 33.2-43.5	M: 43.7-47.0	M: 47.3-50.1	M: 50.2-62.2	
Age, mean (SD)	67 (12)	65 (11)	59 (12)	62 (12)	0.024
BMI (kg/m^2^), mean (SD)	22.9 (3.5)	24.2 (3.7)	24.9 (3.9)	27.7 (4.8)	<0.001
Weight loss (%), mean (SD)	6.0 (6.6)	5.2 (6.2)	5.2 (6.4)	2.7 (5.0)	0.001
Serum-albumin (g/l), mean (SD)	39.9 (7.4)	39.0 (4.0)	39.8 (7.1)	39.5 (5.2)	0.911
Complications^a^, n (%)	12 (30.8 %)	18 (46.2 %)	12 (33.3 %)	12 (27.9 %)	0.329
5-year mortality, n (%)	22 (56.4 %)	25 (64.1 %)	25 (69.4 %)	22 (51.2 %)	0.360

*W* women, *M* men

^a)^ Major postoperative complications within eight weeks after surgery

### Complications

Major postoperative complications were suffered by 115 (31.2 %) patients. The only variable that differed significantly between patients with and without major postoperative complications was serum-albumin concentration (*p* = 0.016). Odds ratio for major postoperative complications with preoperative serum-albumin <35 g/l was 2.08 (*p* = 0.010). There were no significant association between preoperative weight loss and major postoperative complications (*p* = 0.688).

### Overall survival

Preoperative weight loss >5 % (HR = 1.38, *p* = 0.023) and preoperative serum-albumin <35 g/l (HR = 1.52, *p* = 0 .014) were independently associated with reduced overall survival (Table 5). Experiencing a major postoperative complication was independently associated with reduced overall survival (HR = 1.51, *p* = 0.003), but not when patients who died within 90 days after surgery were excluded from analysis (*p* = 0.133) (Table 5).

**Table 5:** Predictors of overall survival, stratified (on disease-categories) analysis

Risk factors, complications and overall survival						
	Unadjusted			Multivariable adjusted		
	HR	95 % CI	P-value	HR	95 % CI	*P*-value
Age >65 years	1.29	0.99-1.68	0.058	1.21	0.92-1.58	0.173
Male gender	1.11	0.85-1.45	0.456	1.02	0.78-1.34	0.873
Weight loss >5 %	1.48	1.12-1.94	0.005	1.38	1.04-1.83	0.023
Serum-albumin <35 g/l	1.80	1.30-2.49	<0.001	1.52	1.09-2.14	0.014
Complications	1.58	1.21-2.06	0.001	1.51	1.15-1.98	0.003
Complications and overall survival excluding patients alive <90 days^a^						
Complications	1.30	0.97-1.75	0.075	1.25	0.93-1.69	0.133
Sarcopenia and overall survival^b^						
Sarcopenia	0.66	0.45-0.98	0.037	0.69	0.46-1.03	0.066
Weight loss, serum-albumin and tissue indices, analyzed as continuous variables, and overall survival^c^						
Weight loss (%)	1.05	1.02-1.08	0.002	1.05	1.01-1.09	0.010
Serum-albumin (g/l)	0.97	0.94-0.99	0.018	0.96	0.93-0.99	0.020
L3 SMI (cm^2^/m^2^)	1.02	0.99-1.05	0.301	1.00	0.96-1.04	0.914
L3 VAT (cm^2^/m^2^)	1.00	1.00-1.01	0.491	1.00	0.99-1.01	0.426
L3 SAT (cm^2^/m^2^)	1.00	0.99-1.01	0.657	1.00	0.99-1.01	0.897

^a)^ Major postoperative complications within eight weeks after surgery. Adjusted for age >65 years, gender, weight loss >5 % and serum-albumin <35 g/l in the multivariable adjusted analysis

^b)^Patients with available preoperative CT images (*n* = 157). L3 SMI <41 cm^2^/m^2^ for women. L3 SMI <43 cm^2^/m^2^ for men with BMI <25 kg/m^2^ and L3 SMI <53 cm^2^/m^2^ for men with BMI ≥25 kg/m^2^. Adjusted for age >65 years, gender, weight loss >5 % and serum-albumin <35 g/l in the multivariable adjusted analysis

^c)^ Patients with available preoperative CT images (*n* = 157). Age >65 years and gender is also included in the multivariable adjusted analysis

L3 SMI below Martin’s cut-off values [26] were associated with statistically significant improved overall survival in the unadjusted analysis (HR = 0.66, *p* = 0.037), but this significance did not remain in the multivariable adjusted analysis (HR = 0.69, *p* = 0.066) (Table 5). Similar results were seen after exclusion of patients without cancer, in both the unadjusted (HR = 0.60, *p* = 0.014) and multivariable adjusted (HR = 0.62, *p* = 0.026) analysis.

There was no significant association between L3 SMI, L3 VAT or L3 SAT and changes in overall survival (Table 5). Preoperative weight loss and serum-albumin, when analyzed as continuous variables in the same subgroup, were independently associated with statistically significant changes in overall survival.

## Discussion

The main aim of our investigation was to explore the novel field of CT-based preoperative body composition indices and compare with traditional indicators for poor prognosis and to explore the significance of non-fatal major postoperative complications. We have shown a significant association between both preoperative weight loss >5 % and serum-albumin <35 g/l and reduced overall survival in a large cohort of patients undergoing major upper abdominal surgery. Conversely, there was no association between L3 skeletal muscle index, L3 visceral adipose tissue index or L3 subcutaneous adipose tissue index, and overall survival. We found an association between major postoperative complications and reduced overall survival, but not when patients who died within 90 days after surgery were excluded from analysis.

The relationship between patient-reported preoperative weight loss and adverse outcome has been known since Hiram Studley’s 1936 publication on mortality after surgery for chronic peptic ulcer patients [4]. The relationship between abnormal serum-albumin and adverse outcome is well documented [2, 3]. Our study has again confirmed the robust significance of these easily available indicators.

CT-based body composition indices constitute a novel field. Two methods for calculating skeletal muscle area have emerged in the recent years: the measurement of the total psoas muscle area and the combined skeletal muscle area on a transverse CT image at the third lumbar vertebrae level [27, 28]. Several different cut-off values for L3 skeletal muscle index (L3 SMI) to define sarcopenia have been suggested, some of them are almost identical. Mourtzakis et al. suggested cut-offs, corresponding to skeletal muscle mass as measured on dual energy x-ray absorptiometry, to be two standard deviations below that of healthy young adults [27]. These cut-offs, 39 cm^2^/m^2^ for women and 55 cm^2^/m^2^ for men, were later used in a consensus document on cancer cachexia [6]. Prado et al. suggested cut-offs in sarcopenic-obese patients at 38.5 cm^2^/m^2^ for women and 52.4 cm^2^/m^2^ for men [29]. Van Vledder et al. suggested cut-offs in patients with colorectal liver metastasis: 41.1 cm^2^/m^2^ for women and 43.75 cm^2^/m^2^ for men [8]. Based on a large cohort of patients with gastrointestinal and lung cancers, Martin et al. developed cut-offs according to Body Mass Index [26]: 41 cm^2^/m^2^ for women, 43 cm^2^/m^2^ and 53 cm^2^/m^2^ for men with BMI under or over 25 kg/m^2^ respectively.

We used the cut-offs suggested by Martin et al. and found a significant association between sarcopenia and improved overall survival in the unadjusted analysis, but not in the multivariable adjusted analysis. We find it unlikely that sarcopenic patients have improved overall survival following major upper abdominal surgery. There was no significant association when L3 Skeletal muscle index was analyzed as a continuous variable, nor was there any difference in five-year mortality between the different quartile categories of L3 SMI index. We suggest that this exemplifies the known hazards of using a dichotomous variable in a heterogeneous cohort of patients [30]. We have indeed presented preoperative weight loss and serum-albumin as dichotomous variables, but the association between these variables and overall survival was confirmed when analyzed as continuous variables. No cut-off values were applied when analyzing L3 VAT and L3 SAT indices. There was no association between these continuous variables and survival and to our knowledge, no cut-off values are established.

The lack of association between the tissue indices analyzed in our study and outcome might indicate that these indices do not necessarily mirror a disease-related catabolic process. Instead, they may provide only a spot measurement of body composition with a large normal variance. In a study with a relatively small number of patients, Awad et al. reported a significant loss of skeletal muscle tissue in a esophagogastric cancer patients who received neoadjuvant chemotherapy, but no association between such tissue loss and clinical outcomes [31]. The potential association between ongoing loss of skeletal muscle tissue, i.e. a catabolic process, and outcomes should be explored further.

An association between sarcopenia and survival in specific patient categories, like primary liver cancer patients or sarcopenic-obese patients has been reported [7, 9, 29]. Tissue indices might be particularly important in these categories, but the number of patients with these characteristics in our study was too small for any meaningful analysis.

The association between sarcopenia and survival may not be equal in all diseases or even in different stages of the same disease. Del et al. reported more complete pathological response and longer progression free survival in sarcopenic, compared to non-sarcopenic, patients with operable breast cancer who received neoadjuvant chemotherapy [32]. Prado et al. reported increased chemotherapy toxicity and shorter time to tumor progression in sarcopenic, compared, to non-sarcopenic patients with metastatic breast cancer who received Capecitabine [11]. Body composition indices might be important when patients receive chemotherapeutic agents that are unevenly distributed in the different body compartments. The reasons behind a low skeletal muscle index might also differ depending on cancer stage and development.

Major postoperative complications have been associated with reduced survival for patients surgically treated for malignant or non-malignant diseases [14–19]. Non-fatal complications (excluding patients who die within 30 days after surgery) has also been linked to reduced survival [14]. This has led to speculations concerning an immune modulating effect of complications which might make the patient more susceptible to cancer recurrence [14, 15]. We extended the definition of fatal complications to death within 90 days and found no association between non-fatal complications and reduced survival. Major postoperative complications may preclude or delay administration of adjuvant chemotherapy [17]. Both the administration and the benefit of adjuvant chemotherapy is diverse in heterogenic upper GI and HPB patient groups like ours and this may also explain why we could not find any association between complications and survival.

Our cohort consisted of patients enrolled in a national multicenter clinical trial [20]. This trial investigated the safety of food at will after all kinds of major upper abdominal surgery [20]. Using this cohort, without further selection of patients, gave us the opportunity to investigate both old and novel indicators of poor prognosis in a clinical setting and to do so prospectively with long observation time.

Our study has several weaknesses: We lacked information about cancer-stage, and the number of patients with available CT-images of sufficient quality was relatively small. The patients in our cohort have not been controlled in a homogeneous follow-up program, so cause of death and disease-specific survival would be unreliable. Despite these weaknesses, our study adds important knowledge: While preoperative weight loss and abnormal serum-albumin were associated with reduced survival independent of disease-categories, the opposite pattern was shown with the body composition indices. Cancer-stage is an important confounder, probably linked to both weight loss and serum-albumin. Exact information about cancer-stage is generally not available in a preoperative setting, which make global indicators, like preoperative weight loss and serum-albumin, important in recognizing advanced disease.

## Conclusion

Our study adds to the core knowledge of preoperative weight loss and abnormal preoperative serum-albumin as important preoperative indicators of poor prognosis. These tools are independent of age, gender, and disease category in upper abdominal surgery. Body composition indices, as defined and utilized in the present study, did not provide additional information of clinical importance. We did not find any evidence of an association between non-fatal complications and long-term survival in major upper abdominal surgery.

### Ethical considerations

Inclusion into the original randomized controlled trial [20], as well as long-term follow-up was approved by the Regional Committee on Research Ethics, Northern Chapter (REK V). All patients provided written consent.

## Abbreviations

GI: Gastrointestinal
HPB: Hepatopancreatobiliary
L3 SMI: Lumbar skeletal muscle index
L3 VAT: Visceral adipose tissue index
L3 SAT: Subcutaneous adipose tissue index
WL: Weight loss
